# Peritraumatic Distress and Its Association with Resilience among a War-Exposed Population: A Cross-Sectional Study

**DOI:** 10.1192/j.eurpsy.2026.11867

**Published:** 2026-07-31

**Authors:** H. K. Yacoub, S. Sabbah, Z. Yacoub, R. Yacoub, Y. Ghannam

**Affiliations:** 1 Faculty of Medicine; 2 Department of Psychology; 3 Department of Mental Health, Al Quds University, Abu Dis, West Bank and Gaza

## Abstract

**Introduction:**

War is an intense traumatic experience that can result in severe psychological distress. Peritraumatic distress (PTD) refers to the emotional and psychological response experienced during or immediately after trauma and is a known predictor of posttraumatic stress disorder (PTSD). Resilience has an important role in coping with trauma-related distress, reducing its intensity, and promoting recovery. Understanding the relationship between PTD and resilience is essential for developing effective mental health interventions in war-affected populations.

**Objectives:**

This study aimed to explore the association between PTD and resilience among a developing population exposed to war-related trauma and to explore the influence of sociodemographic and health-related factors on these psychological responses.

**Methods:**

A cross-sectional study was conducted among 660 Palestinian adults exposed to war-related trauma. Participants completed validated Arabic versions of the Peritraumatic Distress Inventory (PDI) and the Connor-Davidson Resilience Scale (CD-RISC-10). Statistical analyses included descriptive statistics, Pearson correlations, and linear regression models.

**Results:**

Participants reported moderate PTD levels (Mean = 36.1, SD = 9.00), with 16.5% scoring high levels. Resilience scores were also moderate (Mean = 32.35, SD = 9.00), with nearly half (49.7%) scoring in the high range. Higher PTD was significantly associated with higher resilience (B = 0.3, β = 0.301, p < 0.001), a finding that may reflect adaptive coping processes in highly stressful environments. Additional predictors of resilience included age, educational level, employment status, and health condition.

**Table 1.** peritraumatic distress and resilience levels among the participants Table 1. Long description.

Scale	Mean	*SD*	Participants with High score	Participants with Moderate score	Participants with Low score
PDI	36.10	*9.00*	109 (16.5%)	321 (48.6%)	230 (34.8%)
CD-RISK10	32.35	*9.00*	328 (49.7%)	179 (27.1%)	153 (23.2%)

Note. ** p < 0.05

**Table 2.** Correlations between the main study variables Table 2. Long description.

Variables	Peritraumatic Distress	Loss of control and arousal	Negative emotions	Feeling of threat	CD-RISC10
**Peritraumatic Distress**	1	-	-	-	-
Loss of control and arousal	0.805**	1	-	-	-
Negative emotions	0.874**	0.456**	1	-	-
Feeling of threat	0.748**	0259**	0.293**	1	-
**CD-RISC10**	0.301**	0.034	0.347**	0.490**	1

Note. ** p < 0.05

**Conclusions:**

Our findings suggest a significant relationship between PTD and resilience, suggesting that PTD may activate adaptive coping mechanisms during war-related trauma. The study also emphasizes the need for early mental health interventions, resilience-building programs, and targeted support in war-affected regions to control long-term psychological consequences.

**Disclosure of Interest:**

None Declared

## Long descriptions

### Table 1. Long description

The table consists of six columns: Scale, Mean, S D, Participants with High score, Participants with Moderate score, and Participants with Low score.

* The first data row for P D I (Peritraumatic Distress Inventory) shows a Mean of 36.10 and an S D of 9.00. Distribution includes 109 participants (16.5 percent) with a High score, 321 participants (48.6 percent) with a Moderate score, and 230 participants (34.8 percent) with a Low score.

* The second data row for C D - R I S K 10 (Connor-Davidson Resilience Scale) shows a Mean of 32.35 and an S D of 9.00. Distribution includes 328 participants (49.7 percent) with a High score, 179 participants (27.1 percent) with a Moderate score, and 153 participants (23.2 percent) with a Low score.

A footer note indicates that double asterisk p is less than 0.05.

Navigate back to Table 1..

### Table 2. Long description

The table consists of 6 columns and 5 rows of data. The columns are labeled: Variables, Peritraumatic Distress, Loss of control and arousal, Negative emotions, Feeling of threat, and C D - R I S C 10.

Row 1: Peritraumatic Distress correlates with itself at 1.

Row 2: Loss of control and arousal correlates with Peritraumatic Distress at 0.805**.

Row 3: Negative emotions correlates with Peritraumatic Distress at 0.874** and with Loss of control and arousal at 0.456**.

Row 4: Feeling of threat correlates with Peritraumatic Distress at 0.748**, with Loss of control and arousal at 0.259**, and with Negative emotions at 0.293**.

Row 5: C D - R I S C 10 correlates with Peritraumatic Distress at 0.301**, with Loss of control and arousal at 0.034 (not significant), with Negative emotions at 0.347**, and with Feeling of threat at 0.490**.

Note: ** indicates p is less than 0.05.

Navigate back to Table 2..

